# Origins and functions of eosinophils in two non-mucosal tissues

**DOI:** 10.3389/fimmu.2024.1368142

**Published:** 2024-03-22

**Authors:** Katie S. Day, Lucas Rempel, Fabio M. V. Rossi, Marine Theret

**Affiliations:** ^1^ Department of Medical Genetics, School of Biomedical Engineering, University of British Columbia, Vancouver, BC, Canada; ^2^ Department of Biology and Biochemistry, University of Bath, Bath, United Kingdom

**Keywords:** skeletal muscle, tissue repair, immune cells, adipose tissue, eosinophil

## Abstract

Eosinophils are a type of granulocyte named after the presence of their eosin-stained granules. Traditionally, eosinophils have been best known to play prominent roles in anti-parasitic responses and mediating allergic reactions. Knowledge of their behaviour has expanded with time, and they are now recognized to play integral parts in the homeostasis of gastrointestinal, respiratory, skeletal muscle, adipose, and connective tissue systems. As such, they are implicated in a myriad of pathologies, and have been the target of several medical therapies. This review focuses on the lifespan of eosinophils, from their origins in the bone marrow, to their tissue-resident role. In particular, we wish to highlight the functions of eosinophils in non-mucosal tissues with skeletal muscle and the adipose tissues as examples, and to discuss the current understanding of their participation in diseased states in these tissues.

## 1 Introduction

The eosinophil was discovered by Paul Erlich in, 1879, when he observed the distinctive properties of a particular subset of blood leukocytes that exhibited a pink hue when stained with eosin dye (1).

**Figure 1:**
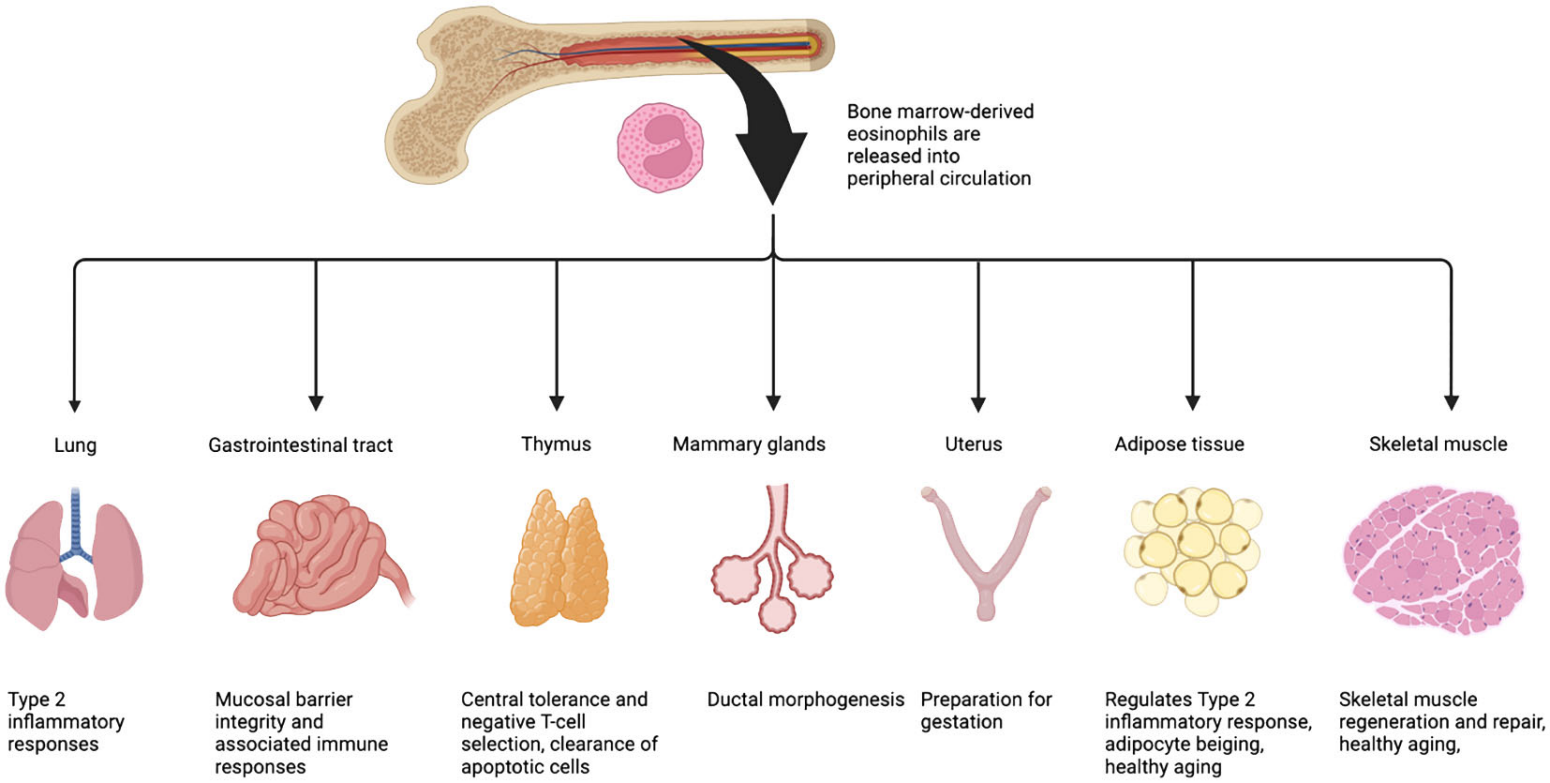
Eosinophils are present in most -if not all- type of tissues. Once fully differentiated, eosinophils exit the bone marrow, enter and patrol the circulation. Upon stimulation (damage, cytokine/chemokine gradient), they migrate to various tissues where they home into the interstitial space and, with the support of other immune cells, provide a Th2 micro-environment that will participate to maintain tissue homeostasis. Eosinophils predominate within the lamina propria of the gastrointestinal tract; the lung parenchyma; the cortico-medullary region of the thymus; the bulbous end of developing terminal end buds of mammary glands; the endometrium of the uterus; and finally, within the interstitial space of the adipose tissue and skeletal muscle. Figure adapted from Marichal et al. (10).

Eosinophils originate in the bone marrow from multipotent hematopoietic stem cells (HSC) that differentiate down the myeloid lineage (2). The common myeloid progenitor (CMP) gives rise to myeloblasts, which are capable of entering granulopoiesis towards one of three types of cells: neutrophils, basophils, and eosinophils (3). The differentiation and maturation of eosinophils along the eosinophilic lineage are dependent on the timely expression and presence of several transcription factors and cytokines (2, 4–6). Mature eosinophils are tissue-resident and distributed among the circulatory and lymphatic system and several organs in the body (2). Within the peripheral blood, eosinophils typically make up less than five percent of all peripheral white blood cells (7–9). Relative to other cell types, eosinophils predominate within the lamina propria of the gastrointestinal tract; the lung parenchyma; the cortico-medullary region of the thymus (in close proximity of CD4/CD8 negative thymocytes); the bulbous end of developing terminal end buds of mammary glands; the endometrium of the uterus; and finally, within the interstitial space of the adipose tissue and skeletal muscle (**Figure 1**). In response to infection or allergen, Type 2 inflammatory (Th2) cells in proximity to the stressor produce high levels of interleukine (IL)-5 which triggers eosinophil infiltration into tissues (11, 12). Pathogenic tissue hyper-eosinophilia arises in a number of diseased states, including atopic dermatitis, allergic airway inflammation, asthma, eosinophilic esophagitis, cancer, and some myopathies such as Duchenne Muscular Dystrophy (DMD) and eosinophilic myositis (13–17).

Mature eosinophils contain granules rich in major basic protein (MBP), eosinophil cationic protein (ECP), eosinophil peroxidase (EPO/EPX), and eosinophil-derived neurotoxin (EDN) (18, 19). Eosinophil granules are cytotoxic and are released in response to bacterial, viral, and parasitic infection as well as allergen exposure (19, 20). Other cell surface markers such as SiglecF (CD170) and IL-5Rα (CD125) allow the study of eosinophils at homeostasis and in diseased states. In human, eosinophils populations are characterized with SIGLEC8, CD62L, CD101 (21), but also CD15 and CD66b (22, 23). However, extensive attempts to study eosinophils across different tissues and diseases have uncovered that these cells are more heterogeneous than initially assumed, both in terms of spatial and temporal expression of various markers and functions (24). Particularly in the lung, blood-circulating eosinophils infiltrate the lung after house dust mite (HDM) stimulation. These eosinophils express higher level of SiglecF, CD34, lower level of CD125, and similar levels of F4/80 and CCR3 compared to resident eosinophils. They also have a highly segmented nucleus and a higher density of granules, suggesting some level of activation. Interestingly, these eosinophil also express CD101, shown to be a marker of non-classical activation, thus they are categorized as inflammatory eosinophils (25). More studies have described different profiles of infiltrating and resident eosinophils after damage, however these are mostly focused on mucosal tissues (21, 26) and very little is known about these subsets in skeletal muscle and adipose tissue.

To resolve this issue, higher-resolution techniques such as single-cell RNA sequencing (RNAseq) have been employed to better understand eosinophil heterogeneity and function. However, due to their poor survival after extraction eosinophils were absent from human and murine single cell RNAseq datasets until recently (27). As a result, the compendium of knowledge on eosinophil function and heterogeneity, particularly those belonging to non-mucosal tissues, is scattered or lacking altogether. The aim of this review is to summarize the origins of eosinophils and discuss the existing evidence for their function in two non-mucosal tissues at homeostasis and in disease.

## 2 Mouse models to study eosinophils

Several mouse models are available to study the role of eosinophils at homeostasis and in disease (**Table 1**). It is important to highlight that these models have systemic changes in eosinophil counts through their lifespan and might have stronger phenotypes than when inducing hyper eosinophilia with IL-5 injection, or when reducing their number with blocking antibody for IL-5.

**Table 1:** List of mouse models to study eosinophils.

Model	Background	Genetic Alteration	Consequence to eosinophils	Original references
ΔdblGATA	C57BL/6J	Deletion of high-affinity GATA-binding site upstream of the GATA-1 promoter	Eosinophil depletion	(28)
	BALB/c		Eosinophil depletion Higher Th2 sensitivity	
IL-5^-/-^	C57BL/6J	Targeted disruption of *IL-5* gene locus	Depletion of CD5+-B1 cells Non-responsive to hyper-eosinophilic stimuli	(29)
EPO-DTA (*PHIL*)	C57BL/6J	Insertion of DTA gene upstream of EPX promoter	Depletion of EOPs and mature eosinophils	(30)
Icsbp^-/-^	BALB/c	Targeted insertion of PGK-neo cassette in exon 2 of Icsbp	Reduced bone marrow eosinophil production Non-responsive to IL-5 stimulation	(31)
IL5-Tg	CBA/CaJ x C57BL/6J	CD3delta regulatory elements inserted upstream to drive T cell expression of IL-5	Hyper-eosinophilia	(32)
	CBA/Ca x C57BL6	IL-5 genomic fragment coupled with the dominant control region of the CD2 locus		(33)
	C57BL6J	Plasmid containing the promoter region of Clara cell secretory protein (CC10) was injected into mouse embryos		(34)
IL4-eGFP (4-get)	C57BL/6J	Transgenic mouse model with IRES-EGFP reporter protein downstream of IL-4 locus	Allow IL-4+ cell tracking	(35)
SiglecF-null	C56BL/6	An R114A point- mutation was inserted in *Siglecf* gene	Show hyper-eosinophilia in case or acute lung damage	(36)
eoCRE	C57BL/6J	Cre recombinase sequence was inserted at the start codon of *Epx*	Allow eosinophil-specific gene targeting	(37)

### 2.1 Eosinophil-deficient models

One of the most common models it the ΔdblGATA mice in which a mutation has been inserted in the double GATA-site 21 base pair upstream of the first exon of the *Gata-1* gene (28). GATA-1 is also needed for the development of red blood cells, megakaryocytes and mast cells, which are not affected in this model due to the specificity of the expression of the targeted exon. It is important to note that this mouse model can be found in two different backgrounds and that it should be compared to its correct control (BALB/c or ​​C57BL/6). Indeed, in comparison to the C57BL/6 mice, BALB/c mice are more sensitive to infectious diseases and allergic reactions, which are both classified as Th2 immune responses. Hence, results coming from either of the backgrounds must be carefully considered.

The IL-5 deficient mouse (C57BL/6-Il5tm1Kopf/J) can also be used to study the absence of eosinophil in diseases. Interestingly, this mouse model does not have a defect in basal eosinophil production, suggesting that IL-5 is not needed for the production of eosinophil progenitors (EOPs) and blood circulating eosinophils. The production of other Th2 cytokines is also not affected, as the levels of IL-4 and IL-13 remain similar to WT controls (29).

A transgenic mouse model expressing diphtheria toxin A (DTA) behind the EPO/EPX promotor can also be used to study the role of eosinophils (PHIL, B6.Cg-Tg(Epo-DTA)#Nal/JleeJ). This results in eosinophil cell death prior to their full maturation and bone marrow exit. In practical terms, this mouse model allows for the depletion of EOPs and mature eosinophils, useful for elucidating the consequences of eosinophil deficiency in development, homeostasis, and disease (30).

Lastly, the interferon consensus sequence-binding protein (Icsbp)-deficient mouse model exhibits reduced bone marrow eosinophil production due to a reduction in the expression of GATA-1 (31). This model is unable to respond to IL-5 stimulation (38).

### 2.2 Eosinophil-abundant models

The IL5-Tg mouse model is used to study the effect of hyper-eosinophilia. In these mice, the gene coding for IL-5 has been placed under a different promotor or contain multiple copies of the *Il5* gene. For example, IL-5 can be overexpressed using regulatory elements from the CD3delta gene to drive T cell expression of IL-5 (B6.Cg-Tg(Cd3d-Il5)NJ.1638Nal/JleeJ) (32), which results in a concentration of 400-800 pg/ml of IL-5 in the serum while it should be undetectable in normal conditions. The sequence coding for *Il5* can also be placed under the dominant control region of the human CD2 promotor (33). Of interest, four independent eosinophilic transgenic lines were established: Tg5C1, Tg5C2, Tg5C3, and Tg5C4 (33). These transgenic lines contain between 8 and 49 transgene copies. Furthermore, IL-5 has been over-expressed under the rat Clara cell secretory protein regulatory elements, expressed in the lung epithelium (B6.Cg-Tg(Scgb1a1-Il5)NJ.1726Nal/JleeJ) (34).

Interestingly, the siglecF-null mice (C.129(Cg)-Siglecftm1.2Avrk/J) display elevated eosinophil infiltration in lung with bone marrow and blood hyper-eosinophilia after ovalbumin (OVA)-induced lung allergy, suggesting that siglecF participates in a negative feedback loop, reducing eosinophil function, and inducing eosinophil apoptosis (36). However McMillan and colleagues showed that the phenotype observed could be due to the experimental procedure (intranasal versus aerosolised OVA) and might be due the extent of the damage induced (39).

### 2.3 Other models

Eosinophil tracking is possible using a GFP reporter under IL-4 promotor: IL-4/GFP-enhanced transcript (4-Get) mouse model (C.129-Il4tm1Lky/J) (35). In order to study the cell-signaling process in eosinophils, a EPX-cre model (eoCRE) was designed by the team of Dr. James J Lee (37). This mouse is a knock-in, with a reduced production of EPX. However, the levels and properties of eosinophils from the eoCRe mice remain unchanged (37).

With multiple mouse models available, eosinophils are now easily targeted and studied. Nevertheless, the choice of model and its background, as well as the type of damage must be taken in account when designing the study.

## 3 Origin of eosinophils: from the bone marrow to the tissue

Once released into the circulation, eosinophils have a very short half-life that can vary from 8 -18 hours in the blood, and up to 6 -7 days once infiltrated in a homeostatic tissue (i.e. non-damaged). For example, in the thymus, eosinophils increase after birth and peak at 2 weeks, after which they fall and rise again during thymic involution (40). In the uterus, eosinophils follow the menstrual cycle as they infiltrate the endometrium adjacent to the myometrium 1 day prior, during, and 1 day after estrus. While these eosinophils are thought to contribute to preparing the uterus for pregnancy, their loss does not impact the fertility of mice (41). In mammary glands, eosinophils are present in and contribute to ductal morphogenesis from 3 - 8 weeks of age (42, 43).

The lifespan of eosinophils can be increased using IL-5, IL-3, or GM-CSF *in vitro*, suggesting that these molecules could also play a role in the viability of eosinophils, as they are released during fundamental processes like tissue remodeling. The infiltration and homing of eosinophils into tissues are driven by several factors (such as eotaxins). IL-5 the eosinophil chemoattractant and pro-survival factor, is necessary for the development and the migration of eosinophils from the bone marrow to the blood (4, 44, 45). Eosinophils are drawn into peripheral tissues via local chemokine attractants (46–48). For example, in homeostatic conditions, Eotaxin-1 (CCL11) is produced by local stromal cells and controls eosinophil migration via circulation to tissues such as the gastrointestinal tract, uterus, mammary glands, and thymus (**Figure 1**) (49–52).

### 3.1 Embryonic development

The role of eosinophils during mouse embryonic development is unclear, as mice lacking eosinophils are viable into adulthood. Moreover, there is very little evidence showing when eosinophils are first produced during embryogenesis. Using the 4-Get mouse model (IL-4 eGFP reporter mouse model) Voehringer et al., showed that a population of GFP+ cells appeared at E14.5, and that cells expressing a putative eosinophil marker pattern such as c-Kit- Sca-1- CCR3- SiglecF+ IL4-eGFP+ arise from the fetal liver at E16.5 (53). While these cells do not express CD34 (expressed by adult bone marrow EOPs), they were able to repopulate the bone marrow after transplantation. *In vitro*, eosinophils can be differentiated from murine embryonic cells cultivated on a feeding layer of OP9 fibroblasts with IL-5, and either IL-3, GM-CSF, or eotaxin (54).

Are eosinophils produced by the Aorta-Gonad-Mesonephros (AGM) or only by the fetal liver? During development, does their appearance follow that of circulating monocytes? While eosinophils are not present in the lung at birth but gradually increase in numbers to reach maximal density by day 7 after birth (25) they are already found in the gut tract before birth. We could then question if there is a sub-population of tissue-resident eosinophils, similar to tissue resident macrophages derived from the yolk sac. The timeline of eosinophil infiltration in non-mucosal tissues such as in skeletal muscle or adipose is unknown, and it would be compelling to see if it corresponds to the development of the microbiota, as suggested for lung infiltration (25).

### 3.2 Bone marrow hematopoiesis

**Figure 2:**
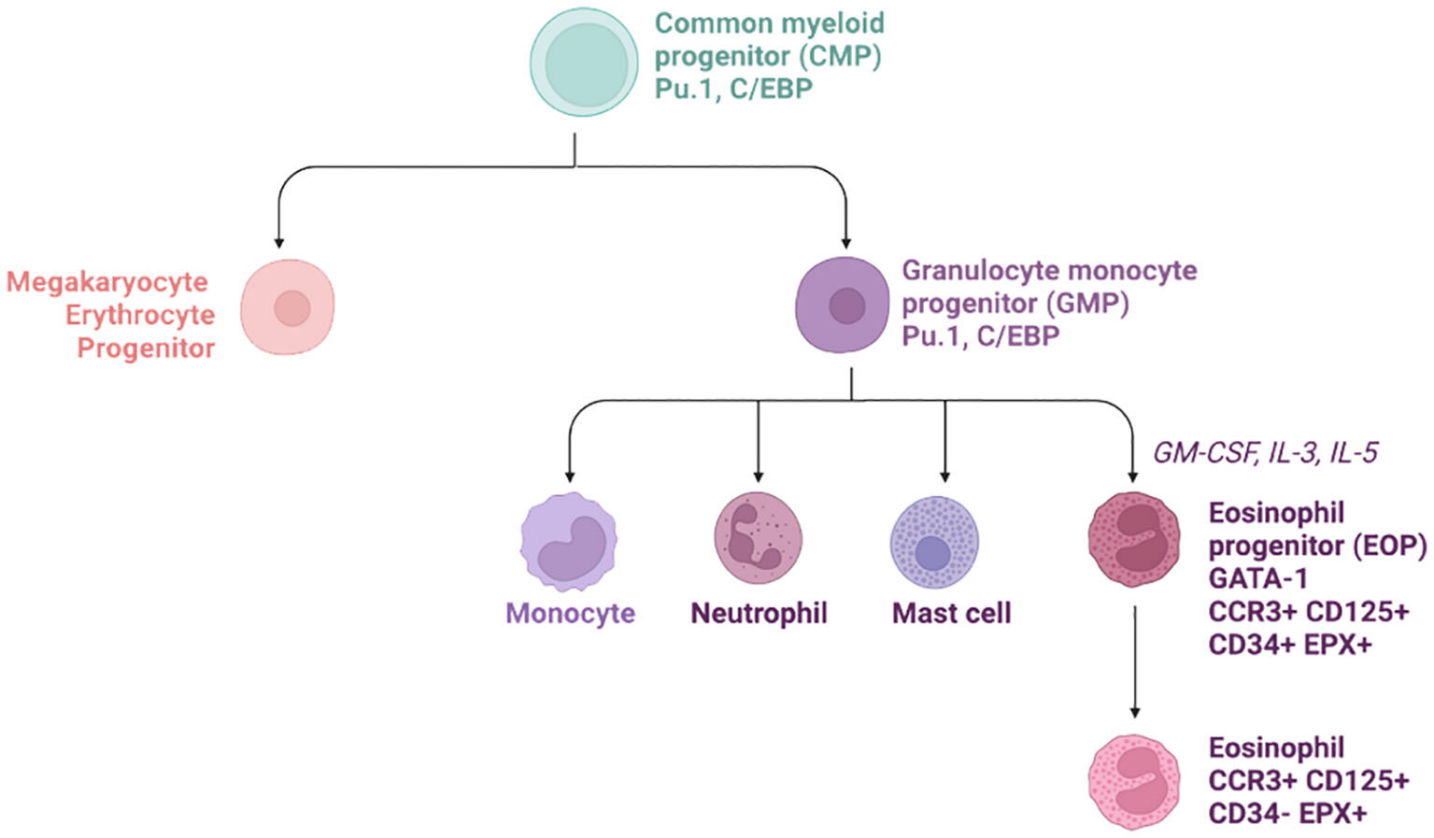
Hierarchical tree of eosinophil progenitor formation in the bone marrow. The common myeloid progenitor (CMP) give rise to both megakaryocyte Erythrocytes Progenitors (MEP) and Granulocyte monocyte progenitor (GMP). The latest then produce the eosinophil progenitors thanks to various chemokines such as GM-CSF, IL-3, and IL-5. Later on, these cells down-regulate CD34 and leave the bone marrow for the circulation.

During adulthood, eosinophils are released from the bone marrow into the circulation and their production depends on both transcription factor activity and cytokine stimulation (**Figure 2**).

A key transcription factor required for eosinophil production is PU.1. PU.1 is an ETS family member and is only expressed in hematopoietic cells. It is involved in regulating the balance between the lymphoid and myeloid lineage. PU.1-deficient mice lack B cells and dendritic cells, but also all cells belonging to the myeloid lineage such as monocytes and neutrophils. PU.1-deficient mice die within the first 48 hours post birth due to the absence of the wide range of immune cells (55). C/EBP (CCAAT/enhancer-binding protein) is expressed in CMPs and its activation leads to increased myeloid and eosinophil differentiation (56). Interestingly, deletion of C/EBPα will only affect the myeloid lineage, suggesting there are differential roles of C/EBPα/β in eosinophil development (56). GATA-1 (a zinc finger family member) is expressed at a later stage of the eosinophil differentiation as it regulates the expression of CCR3, CD125 (IL-5Rα) and other mature granule protein genes such as MBP, EPX, ECP, EDN, and Charcot-Leyden crystal protein (CLC, Galectin-10) (28).

Interestingly, ICSBP (an IFN-g induced transcription factor) has also been shown to be involved in eosinophil differentiation as it regulates GATA-1 expression. *In vitro*, HSC from ICSBP-KO mice do not respond to IL-5 stimulation, leading to a defect in the production of eosinophil progenitors (38).

Id proteins are also implicated in eosinophil development. Id proteins are composed of 4 isoforms and are basic helix-loop-helix transcription factors that lack a basic DNA binding domain. Their function is essential for stem cell fate, as they can block the differentiation of progenitors, promoting their proliferation and delaying senescence. In regard to eosinophil differentiation, Id1 and Id2 have opposing roles. Id1 inhibits eosinophil development whereas Id2 accelerates the final maturation of eosinophils. The roles of Id3 and Id4 in eosinophil production have not been described (57)

Finally, Friend of GATA (FOG) acts as a repressor of the eosinophil lineage. FOG is also part of the zinc finger family and binds to the N-terminal finger motif of GATA-1, inhibiting its ability to activate the transcription of *Mbp* (58).

Once committed to the eosinophilic lineage, the final differentiation step requires the synergic stimulation of IL-3, GM-CSF, and finally, IL-5. While IL-3 and GM-CSF enhance myeloid differentiation, IL-5 is specific to EOPs due to its unique affinity to CD125 (IL-5Rα). Elevated levels of IL-5, either from intravenous cytokine injection, or in a genetic mouse model over-expressing IL-5 under the CD3 promoter, induce blood hyper-eosinophilia (**Table 1**). Fully differentiated eosinophils are released from the bone marrow into the circulatory system, until they reach their tissue of interest.

### 3.3 Eosinophil homing and homeostasis

Eosinophils do not remain in the blood to patrol for pathogenic organisms, but rather use it as an expressway to go from the bone marrow to tissues of interest. These include primarily mucosal tissues such as the lung or gut. To enter tissues, eosinophils primarily rely on a CCL11 concentration gradient across epithelial cells, endothelial cells, or fibroblasts, as well as other chemokines such as eotaxin-2 and 3 (CCL24 and CCL26), via CCR3. While these cytokines are the main regulators of eosinophil infiltration, it is interesting to note that CCR3, the receptor for CCL11, has been shown to induce a negative feedback loop on eosinophil response to inflammatory stimuli. For example, pre-treatment with the chemokine Monokine induced by gamma interferon (Mig, also known as CXCL9) inhibits eosinophil responses by a CCR3-Rac2 dependent mechanism (59).

Once in the tissue, eosinophils protect against parasitic infections (in the gut), react to allergens (in the lung) or maintain a Th2 environment (in the adipose tissue). Their different functions suggest levels of heterogeneity within tissues, which have been difficult to study as eosinophils half-life can be a short as a few hours. The first scRNAseq eosinophil dataset was conducted by Gurtner et al., where the authors separated eosinophils from bone marrow, blood, spleen, stomach, small intestine, and colon into their progenitor, immature, circulating, basal, and active forms (27). Most importantly, they showed that two types of eosinophil exist within the GI tract as basal (i.e. non-activated) and activated populations. In the GI tract, these two populations can be separated by the expression of PD-L1 and CD80, with the activated population expressing both markers. PD-L1 and CD80 expression is regulated by NF-kB signaling, and *in vitro* IL-33 stimulation was sufficient to induce eosinophil activation. scRNAseq was also performed on the IL-5-Tg mice, which demonstrated that there was a population of pre-activated eosinophils in the absence of any prior damage or stimulus (27). The authors further validated their findings on WT C57BL/B6J mice. The role of this activated population has been further studied in colitis and inflammatory bowel disease, but not in other tissues thus far. It is imperative that the existence and the role of this population is confirmed in other eosinophil-rich tissues, such as in the lung and in some non-mucosal tissues.

## 4 Role of eosinophils in skeletal muscle homeostasis

Skeletal muscle is one of the three types of muscles found in the body and is the only type under voluntary control. This highly organised striated tissue is composed of multinucleated myofibers that produce contractile forces used to generate locomotion and stability (for review of its structure please read (60)). At homeostasis, skeletal muscle stem cell (also called satellite cells (SCs)) turnaround is slow to non-existent. However, after damage, skeletal muscle is able to regenerate and repair itself thanks to a complex cell-cell crosstalk. Muscle regeneration occurs through a multi-step process that includes: necrosis/degeneration, inflammation, maturation, and functional recovery (61). Skeletal muscle homeostasis and myofiber regeneration are affected in various pathological conditions such as in aging (sarcopenia), cancer (cachexia) and various myopathies such as Duchenne Muscular Dystrophy (DMD), Limb-girdle muscular dystrophies (LGMD), or eosinophilic myositis (for review read (62)). It is widely documented that eosinophils play an active role in Th2 immune responses in a variety of tissues and disease states, but the role that eosinophils play and the underlying mechanisms of activation and signalling in skeletal muscle tissue remains largely unknown.

### 4.1 Acute damage

**Figure 3:**
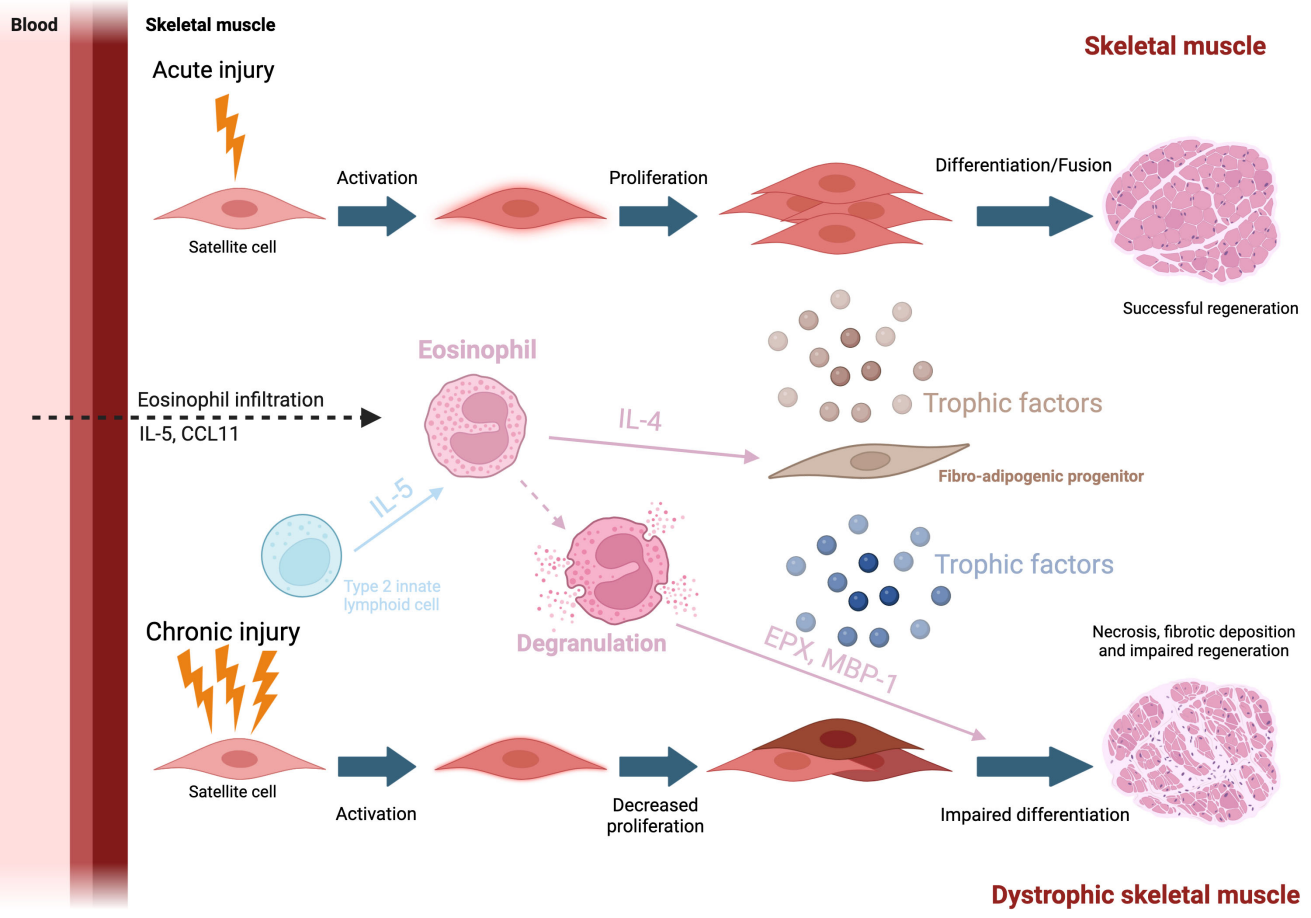
Eosinophil and muscle repair Top: After an injury, eosinophils are attracted to the site of damage via the production of CCL11 and IL-5. They then secrete various cytokine and in particular IL-4, which will support FAP trophic function toward myogenic cells. Bottom: In case of chronic injury, such as seen in dystrophic muscle, eosinophil will degranulate Major basic protein 1 will be released in the environment, lysing myofibers, participating into the degenerative phenotype.

Acute injury occurs in skeletal muscle either when the muscle experiences a traumatic or contraction-induced injury to the muscle fibres. Immune cells, and in particular macrophages have been shown to play a vital role in the repair process after acute injury (for review read (63)). In the first few hours after injury, tissue-resident cells produce CCL2, a chemoattractant to signal and recruit various inflammatory cells to the site of injury (64–66). At the site of injury, Ly-6C^hi^ macrophages express pro-inflammatory cytokines, further promoting the recruitment of monocytes and eosinophils, which in turn, promotes myogenic cell proliferation (67, 68). Macrophages then transition to a pro-regenerative phenotype and down-regulate Ly-6C. Ly-6C^low^ macrophages produce TGF-β, IL-10, and IGF-1 which have been shown to stimulate myogenic cell differentiation and promote muscle growth (69–71). While macrophages have been studied for many years, it is not before 2013 that eosinophils started to be studied in the context of muscle regeneration. Heredia and colleagues found that the BALB/c ΔdblGATA mice (deficient in eosinophil, **Table 1**) exhibited delayed skeletal muscle regeneration, suggesting that factors secreted by eosinophils may play a role in skeletal muscle regeneration (72) (**Figure 3**). Further experiments show that after acute injury, a lack of Th2 cytokines (i.e. IL-4 and IL-13) showed significant delays in skeletal muscle regeneration, with the formation of adipocytes suggesting that eosinophil protect muscle against intramuscular adipose tissue (IMAT) (72). Following this, we have investigated the effects of hyper-eosinophilia on muscle repair in acute damage conditions by using the IL5-Tg mice. Significant decreases in the number of myonuclei were found at 7 days post injury, and decreased myofiber size at 14- and 28- days post injury, suggesting that skeletal muscle hyper-eosinophilia impacts the ability of skeletal muscle to regenerate (73). Furthermore, we have shown that during skeletal muscle regeneration eosinophils do not only produce Th2 cytokines but can also produce various molecules that have been shown to affect myofiber stability (please see below in DMD). Similar to macrophages during skeletal muscle regeneration, eosinophils need to be regulated at two different levels: number and inflammatory profile. Acute damage is often associated with pain. Of interest, IL-5 has been associated with pain in fibromyalgia. LPS stimulated PBMC from fibromyalgic women release more IL-5, along with IL-4 and IL-2, than pain free women (74). While no increase in IL-5 was observed in a mouse model of fibromyalgia (acidic saline injected in the *gastrocnemius*), i.v. injection of IL-5 improved the mechanical thresholds of the paw, sign of a decrease in pain (74). This treatment has since then been further explored with behavioral experiments, however only monocyte and T cell populations were analyzed and no eosinophil phenotyping was done on muscle tissue (75). When investigating intramuscular levels of cytokines in patient with jaw muscle pain, IL-5 was under the limit of detection (0.49 pg/mL) and removed from the study, along with IL-2, IL-4, IL-10 and IFNγ, suggesting that the increase in IL-5 associated with pain could be a systemic response (76).

### 4.2 Aging

The extent to which skeletal muscle can regenerate is dependent on the body’s ability to maintain its muscle-resident stem cell population. When the body ages, changes in the microenvironment and the niche of the SCs contribute to a decreased regenerative capacity of skeletal muscle (77). In addition, aging results in sarcopenia which is the involuntary loss of lean muscle mass (atrophy) with a reduction in force production and function. Sarcopenia is associated with a loss of SC activation, differentiation, and fusion, which consequently delays the capacity for muscle to repair itself. This can be exacerbated by other factors including muscle trauma and disease. An increase in blood eosinophil numbers have been associated with aging in the general population and in diseases such as asthma, type 2 diabetes (T2D), and cardiovascular diseases (49, 78, 79). However, with respect to aged skeletal muscle, research has mainly focused on age-related regenerative failures in both SCs and Fibro adipogenic progenitors (FAPs) (77, 80). FAPs are a type of tissue-resident mesenchymal stromal cell (MSC) that can be found by the SC niche and are known regulate muscle homeostasis and myogenesis (81, 82). Aberrant FAP activity may contribute to fibrotic deposition and adipogenesis (83). Some studies have looked at age-related changes in the inflammatory response to injury (inflammaging) but so far no study has explicitly researched the role of eosinophils in aged skeletal muscle (84). The Mathis laboratory has showed that during regeneration, FAPs are the main source of IL-33, a potent Th2 alarmin. In aged muscles (>6-month-old mice), IL-33 production is decreased, resulting in a decrease in regulatory T cell (Treg) accumulation, and a delay in muscle regeneration (85). Interestingly, the authors also showed a decrease in eosinophil number in aged muscle which could be also due to a decrease in IL-33 production, and potentially a decrease in type 2 innate lymphoid cell (ILC2) number, which has not been explored yet. As many cells express ST2 (IL-33R, *Il1rl1*), total KO display strong delay in muscle regeneration. In particular, the Kronke lab showed that IL-33 was required for macrophage skewing and proper alternative polarization during muscle regeneration (86). Neither the number of eosinophil nor their activation level were explored in this ST2^-/-^ model. Age-related changes in eosinophils have been explored in other tissues than skeletal muscle. In asthma patients, aging has been associated with a decrease in eosinophil “effector” function which is implicated in physiological processes such as tissue remodelling, immunomodulation and cellular interactions, and degranulation (process of releasing granule proteins). This which can be toxic to tissue resulting in damage and increased inflammation (79). Recently, a study looked at age-related changes in eosinophils in skeletal muscle in T2D patients and investigated the relationship between inflammation markers (including eosinophils) and skeletal muscle mass in patients over 60 years of age (87). The study determined that eosinophils were positively associated with sarcopenia progression in older patients with T2D. Sarcopenia is a mortality risk factor for older individuals especially when comorbid with T2D, meaning sarcopenia prevention is an important therapeutic goal in human health (87). However, the study could not determine the precise role that eosinophils play during the development of sarcopenia. Furthermore, eosinophil number and muscle atrophy were not correlated in younger patients with T2D, suggesting that age is a key parameter in eosinophil function.

### 4.3 Duchenne Muscular Dystrophy

Duchenne Muscular Dystrophy (DMD) is a X-linked genetic disease characterised by muscle weakness and progressive degeneration due to a mutation found on the *DMD* gene that encodes for the DYSTROPHIN protein (Hoffman et al., 1988). Mutations in the DMD gene can either result in the formation of an incomplete, unstable form of DYSTROPHIN, to a complete absence or to the expression of very low levels of DYSTROPHIN as seen in BMD (Becker Muscular Dystrophy). Other forms of myopathies (limb-girdle) are linked to mutations in the components that form a protein complex associated with Dystrophin (Blake et al., 2002). In the early, 1980s, the single KO DMD^mdx^ (*mdx*) mouse was discovered from a C57BL/10ScSn colony. It showed elevated muscle creatine kinase levels and histological characteristics of muscular dystrophy: increase in necrotic muscle tissue, phagocytosis and fibrosis (88). The initial round of muscle degeneration is first observed around the 3-4 week mark in *mdx* models (89, 90). In contrast, fibrosis, a hallmark of the disease, may take longer to develop, and deposit are first seen in the diaphragm. Hence the *mdx* model is not able to replicate all symptomatic aspects of DMD progression in humans. To improve upon this, the model was crossed with other genetic backgrounds and with other mutations (for example with the Utrophin^-/+^ mice) to better mimic the phenotypic characteristics that are seen in the progression of DMD in humans (91–93). In recent years, *mdx* models lacking eosinophils (*mdx*:PHIL) or inducing hyper-eosinophilia (*mdx*:IL-5tg) have been developed to understand their role in DMD. While eosinophils are one of the lesser studied leukocytes in skeletal muscle, they have been shown to infiltrate skeletal muscle tissues in both human DMD patients and *mdx* mice (15, 94, 95) Once infiltrated, eosinophils degranulate and release cytotoxic granule proteins (including EPX and MBP-1) that lyse myofibers causing additional damage in *mdx* mice (94) (**Figure 3**). Interestingly, eosinophil depletion (using anti-CCR3 antibody) reduced muscle damage in *mdx* mice, however, the genetic ablation of MBP-1 in *mdx* mice yielded mixed phenotype as it reduced collagen deposition, but did not improve skeletal muscle repair. These conflicting results suggest both MBP-1 dependent and independent mechanisms (96). The authors suggest a mechanism in with depletion of MBP-1 increases cytotoxic CD8 T cells activity and a possible switch of the inflammation towards a strongly Th1 response (although with no changes in the expression of *Nos2*, *Tnfa*, and *Ifng*). Moreover, treating *mdx* mice with prednisolone, an FDA approved corticosteroid used to treat DMD in humans, strongly decreases eosinophil number in *mdx* skeletal muscle. Interestingly Sek and colleagues observed slight changes in levels of IL-4 in skeletal muscle in mice strains with affected eosinophil infiltration (mdx, mdx:PHIL, and mdx:IL5Tg) but found no correlation between the amount of IL-4 and the extent of eosinophil infiltration (97) suggesting a potential role for another IL-4 producing cell, such as ILC2s. The authors also found no differences in muscle repair between eosinophil-sufficient (*mdx)* and eosinophil-deficient (*mdx:*PHIL) mice at an early age (97). In contrast, we found that hyper-eosinophilia had a significant effect on muscle fibrotic deposition and muscle repair (73). Notably, technical differences can be found between the studies, such as the background of the mice (C57BL/6J vs DBA) or the experimental endpoint (4 weeks vs 6-12 months), which could explain the conflicting results. In particular for the mdx:IL-5Tg mouse model, long term systemic hyper-eosinophilia could have led to confounding effects from other tissues such as the lungs. Of note, it has been recently shown that ILC2s present in skeletal muscle may regulate eosinophils in muscular dystrophy. Kastenschmidt and colleagues found elevated levels of eosinophils and IL-5 in both DMD patients and *mdx* mice (15). The authors also showed that by depleting IL-13-producing cells (which include ILC2s) in *mdx* at 4 weeks of age, IL-5 levels were decreased and muscle eosinophilia was diminished, suggesting that IL-13 is required for eosinophil infiltration (15). This phenomenon is well known in other tissues such as in the gut or adipose tissue but was not described in the muscle until then (98, 99). Collectively, these studies suggest that eosinophils may play a more important role in the later stages of DMD, proposing an alternative method of skeletal muscle eosinophilia via ILC2 regulation.

## 5 Role of eosinophils in adipose tissue

Adipose tissue, like skeletal muscle, is highly vascularized, permitting access for circulating immune cells, such as eosinophils, to maintain adipose tissue homeostasis (49, 100). Both circulating and tissue-resident eosinophils play a role in regulating the typical functions of adipose tissue, energy storage, metabolism, and endocrine function (49, 101). Shah et al. have performed mRNA sequencing from blood eosinophils, and resident eosinophils from two white adipose tissue (sub-cutaneous and gonadal) (102). While major changes were observed between the blood eosinophils and the tissue resident populations, such as upregulation in the IL-4/IL-13 signaling pathways, very little transcriptomic changes were observed between the two types of adipose tissue, and no pathways were significantly changed (102). This suggest that white adipose resident eosinophils do not differ between locations and that very little heterogeneity is observed within different white adipose tissues. Interestingly, while anti-IL5 therapy (using Mepolizumab, Reslizumab, or Benralizumab) in patients with asthma decreased their BMI (103), blood hyper-eosinophilia was associated with a decreased risk of T2D in an adult cohort composed of both women and men in China (104). Suggesting that the role of eosinophils in human health seems to depend on the disease category.

**Figure 4:**
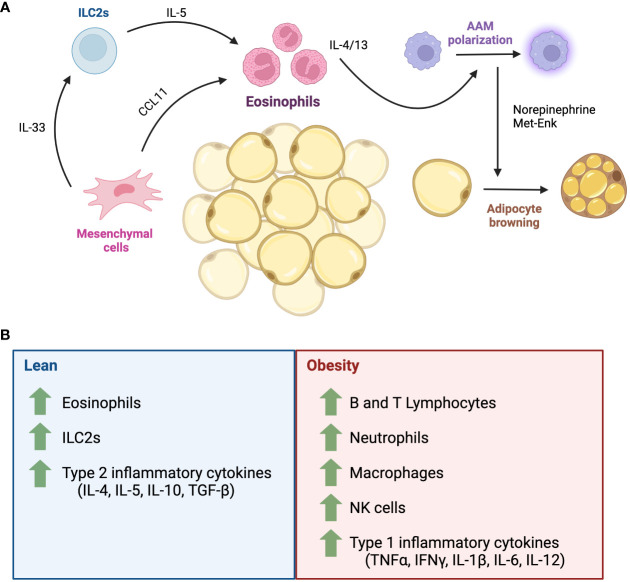
The role of eosinophils in adipose tissue homeostasis. **(A)** Adipose tissue is composed of a rich network of adipocytes, stromal cells and immune cells. In adipose tissue homeostasis, the eosinophil population is maintained by signals from mesenchymal stromal cells and ILC2s. Eosinophil-secreted IL-4 and IL-13 maintains alternatively activated macrophage polarization, which produce catecholamines that promote thermogenesis via the biogenesis of beige adipocytes. **(B)** Major inflammatory cells in lean and obese states. AAM, alternatively activated macrophages; CCL11, eotaxin-1; IL, interleukin; ILC2, Type 2 innate lymphoid cell; IFNγ, interferon gamma; Met-Enk, Met-enkephalin; NK, natural killer cells; TNFα, tumour necrosis factor alpha; TGF-β, transforming growth factor beta.

Eosinophil functions are thought to be established by a MSC-ILC2-eosinophil axis, whereby adipose tissue MSCs produce IL-33 to maintain ILC2 and eosinophils, in turn, ILC2s and MSCs respectively secrete IL-5 and CCL11 to maintain the eosinophil population (**Figure 4A**) (105). Among eosinophil secreted factors, IL-4 and IL-13 are important for adipose tissue macrophage polarization. Alternatively, activated macrophages induced by these factors release catecholamines that contributes to thermoregulation through promoting lipolysis of white adipocytes and promoting the expression *Ucp1* in brown adipocytes. These changes contribute to an elevated energy expenditure and increased thermogenesis (49, 99, 106–111). Furthermore, eosinophil-produced IL-4 can minimize local and systemic age-related changes by attenuating elevations in Th1 cytokines such as IL1-β in aged mice, favouring a Th2 environment (**Figure 4B**) (49). In contrast, Th1 cytokines are also elevated in adipose tissue during obesity, and an Th2 inflammatory environment has been linked to the promotion of the lean/non-obese state (49, 109, 112–114). Due to their role in adipose tissue homeostasis and as orchestrators of the Th2 response, eosinophils and their immune responses have been investigated in hopes of unveiling a mechanism whereby they may confer protective effects against metabolic diseases like obesity and diabetes.

### 5.1 Obesity

One of the characteristics of obesity is a low-grade systemic inflammation, where the abnormal expansion of hypertrophic adipocytes results in the secretion of adipocyte-derived metabolic factors, infiltration of Th1 inflammatory immune cells (**Figure 4B**) such as Ly-6C+ monocytes/macrophages, neutrophils, Natural Killer cells and T Helper 1 cells (112). These cells release pro-inflammatory cytokines such as IFNγ, TNFα, IL-6, IL-12, among others, which can lead to adipose tissue damage and abnormal function (99, 112, 115). Whether eosinophils and Th2 response can oppose this process and promote a metabolically “lean” state has been a controversial topic of discussion. Indeed, high fat diet (HFD)-treated mice have elevated levels of IFNγ (116), which can repress IL-33-mediated ILC2 activation (117) and IL-33^-/-^ mice spontaneously gain weight (118). C57BL/6 mice fed on an 10-14-week HFD, or those genetically predisposed (C57BL/6 ob/ob) also have reduced levels of perigonadal adipose eosinophils (101). Eosinophil-deficient BALB/c mice (ΔdblGATA) fed on a 15-week HFD gained more weight than eosinophil-competent mice, which was attributed to an increase in perigonadal adipose tissue mass. Meanwhile, hyper-eosinophilic IL-5Tg mice on a BALB/c background have smaller depots of visceral adipose tissue (101). IL-5 deficient mice fed on an 18-20 week HFD gained more weight, have increased total body adiposity, and perigonadal adipose weight compared to IL-5 sufficient mice (98). Contrary to these findings, Lee et al., 2018 showed that C.129S1(B6) ΔdblGATA mice fed on a HFD gained less weight and showed reduced body fat, smaller enlargement of adipocytes and decreased expression of adipogenic genes such as *Retnlg*, *Alox15*, and *Drd2* (119). However, ΔdblGATA mice (C.129S1(B6)-Gata1tm6Sho/J) were more insulin resistant, with increased lipid storage in the liver compared to BALB/c WT mice (119). Furthermore, they found that eosinophils migrate towards adipocytes in a CCL11-dependent process *in vitro*. This was supported *in vivo* with the WAT of BALB/c WT HFD mice having an increased frequency of infiltrating eosinophils, in concordance with an increased expression of WAT *Ccr3* and *Ccl11* (119). These obesity studies reported that male mice were used to conduct experiments, herein lies an area where new knowledge can be unveiled with study of female mice. Indeed, it has been demonstrated that female BALB/c mice exhibit a stronger STAT6-dependent Th2 response and increased eosinophilia to triggers such as ovalbumin (120), compared to male mice. Thus, future studies should examine whether sex influences the impact of eosinophils and Th2 environment on obesity. Moreover, all the mice were not all from the same background (BALB/c or C57BL/6) which could have contributed to the conflicting results between Molofsky and Lee. In humans, the role of eosinophils in adipose tissue health and its metabolic regulation remains largely conflicting. According to Brigger et al., the number of eosinophils in adipose tissue is negatively correlated with age (49). Other studies have reported a positive correlation between blood eosinophil count and body mass index in obesity, however this was not as sticking as with the number of neutrophils (121), while metabolic syndrome was associated with higher eosinophil count in subcutaneous white adipose tissue (122). The overall profile (inflammatory vs resident) of eosinophils in these studies was not assessed.

### 5.2 Metabolism

Brown and beige adipose tissues can counteract obesity by metabolizing sugars and fats, releasing the excess energy as heat (123). However, an obese state has been shown to promote the transition of adipocytes from beige-to-white, impacting energy balance and promoting weight gain (123, 124). Eosinophils have been demonstrated to regulate beige adipocyte formation though a variety of mechanisms, including white adipocyte lipolysis and brown adipocyte thermogenesis, which has implications for energy metabolism and weight gain (109, 117, 125). Indeed, eosinophils produce IL-4 abundantly in subcutaneous and perigonadal visceral white adipose tissues (109). Qiu et al. showed that the loss of eosinophils (in the 4-Get- ΔdblGATA mice), or IL-4/13 signaling (BALB/cJ IL-4/13^-/-^), impaired cold-induced biogenesis of beige fat from subcutaneous white adipose tissue in mice, as assessed by *Ucp1* expression and UCP1 protein quantification (109). In addition, administration of exogenous IL-4 to thermoneutral mice increased beige fat mass, and eosinophil-derived IL-4, along with IL-13 from ILC2s was demonstrated to directly promote the differentiation of PDGFRα+ adipocyte precursors (MSC-like cell) to beige adipocytes, rather than white adipocytes (109). Exposure to cold temperature also induces the production of fibroblast growth factor 21 (FGF21), which act as an autocrine loop to induce CCL11 secretion by fibroblast and promoting eosinophil infiltration in WAT (117). Alternatively, eosinophil-derived IL-4/IL-13 has been linked to the polarization of adipose tissue macrophages towards a Th2 (Ly-6C^-^) responsive phenotype. Such macrophages increased tyrosine hydroxylase expression and increased catecholamine synthesis, which in turn promoted the formation of beige adipocytes (109, 111, 126) although this has been debated (110). In addition, eosinophils themselves have been explored as synthesizers of catecholamines which also could promote beige fat formation (127).

Thus, existing evidence withstanding the contributions of eosinophils in adipose tissue metabolism has yet to be fully understood. Future studies will be required to unravel their precise role.

### 5.3 Type 2 diabetes

Obesity and T2D are frequently co-morbid, with obesity being the leading risk factor for developing T2D (128). The chronic excess intake of glucose and fats can stimulate the release of Th1 cytokines (such as TNF-α, and IL-1β) and activate Th1 responsive inflammatory cells in adipose and pancreatic tissues such as Ly-6C+ macrophages and Th1 helper T cells (115, 129, 130). Whether eosinophils and their role in Th2 inflammation could counteract the development of T2D is a question posed by some researchers. As reported by Lee et al., BALB/c ΔdblGATA mice fed on an HFD have been observed to be insulin resistant and have poor glycemic control (119). In a study done by Bolus et al., recombinant IL-5 administered to obese mice on an 8-week HFD did not rescue metabolic parameters such as blood glucose, plasma triglycerides, free fatty acids, or cholesterol (131). On the other hand, numerous studies have examined the effect of the Th2 response on metabolic parameters in obese mice fed an HFD. In order to induce a Th2 response, Hams et al., injected HFD-fed C57BL/6J mice with recombinant helminth egg-derived w1 protein. Overall these mice did not gain weight as much as control, had smaller adipocyte hypertrophy, and displayed adipose tissue hyper-eosinophilia and increased numbers of other Th2 cells (such as ILC2s) and IL-33 (132). Another study showed comparable results in white adipose tissue (133). The role of eosinophils in diabetes in humans has been less studied. However, in a study on Chinese populations, T2D prevalence and blood eosinophils were inversely correlated (104), confirming that eosinophils might have a protective role against the disease. On the other hand, Sokolova et al. reported in, 2017 that non-obese patients with T2D exhibited higher levels of Th2 cytokines (IL-4, IL-5) (134).

A positive correlation between increased levels of Th2 cytokines and eosinophil number is not always true. Interestingly, Brigger et al. showed that while aged C57BL/6J mice (18-22 months) produced higher levels of the chemokine CCL11 in epididymal fat, the number of adipose tissue eosinophils decreased (49). This was complemented by increases in Th1 cytokines TNF-α, IL6, and IL1-β in aged mice. Furthermore, when aged mice were connected to young (2-3 months) mice of the same background by parabiosis, eosinophil number and Th1 cytokines were restored to WT levels, with eosinophils from the young donors taking residence in the epididymal fat of their older partner (49). Given that the prevalence of obesity and diabetes increase with age (135) perhaps the ability of adipose tissue eosinophils to regulate inflammation associated with metabolic diseases like obesity and diabetes is diminished over time and at a systemic level.

Overall, while our understanding of the role circulating and tissue-resident eosinophils play in maintaining adipose tissue homeostasis is well-established, the exact role of eosinophils in metabolic diseases such as obesity and diabetes demands further investigation before we can begin working towards eosinophil-based therapies.

## 6 Discussion

Overall, eosinophils are a very well-studied cell type, in particular in the lung and in the intestine where two populations of eosinophil have been described after damage: the inflammatory eosinophil (iEos) and the resident eosinophils (rEos). Knowing that the half-life of eosinophil in the tissue is 2 to 6 days, we could ask whether rEos are true tissue-resident cells like macrophages. Circulating eosinophils are thought to infiltrate after stimuli/damage and participate into the inflammatory response. However, most of the studies have been carried out in an acute setting with very little return to homeostasis by the experimental end-point, which could potentially show that inflammatory eosinophils eventually become similar to pre-damaged resident eosinophils. In that case, we could question whether these differences represent true heterogeneity, or if they are rather similar to the relationship between monocytes and macrophages. Longer experiments, associated with bone-marrow transplantation or parabiosis to follow their infiltration may help answering these questions. Moreover, eosinophil function and heterogeneity in muscle homeostasis is still to debate. Indeed, as eosinophils do not seem be to principal actors of muscular diseases, the comprehension of their behaviour is not the focus of the muscle community. However, as any immune cell, their function can be modulated by cues and tuning their level of activation could have positive effect on muscle resident cells such as FAPs, myogenic cells, and myofibers. Furthermore, are there skeletal resident eosinophils? What is their half-life? Do infiltrating eosinophils display a similar profile to iEos in the lung or the gut? Flow cytometry coupled with scRNAseq or CITE-seq at various time point after injury on parabiotic pairs would be a way to answer these questions. In the adipose tissue, eosinophils have been more studied due to the obvious change in number during weight gain, systemic inflammation, age, and T2D and are now proposed as key players when it comes to adipocyte health. As skeletal muscle health is also affected by the various parameters cited above, it would not be surprising if eosinophils present in skeletal muscle act similarly. In particular, the role of eosinophil in the development of skeletal muscle insulin resistance has not been investigated. As of now, the results within the muscle community are conflicting and a better characterization of these cells would help to understand their function, with potentially better clinical applications.

## Author contributions

KD: Writing – original draft, Writing – review & editing. LR: Writing – original draft, Writing – review & editing. FR: Writing – review & editing, Resources. MT: Conceptualization, Supervision, Writing – original draft, Writing – review & editing.
